# Multiplex sequencing of bacterial artificial chromosomes for assembling complex plant genomes

**DOI:** 10.1111/pbi.12511

**Published:** 2016-01-23

**Authors:** Sebastian Beier, Axel Himmelbach, Thomas Schmutzer, Marius Felder, Stefan Taudien, Klaus F. X. Mayer, Matthias Platzer, Nils Stein, Uwe Scholz, Martin Mascher

**Affiliations:** ^1^ Leibniz Institute of Plant Genetics and Crop Plant Research (IPK) Gatersleben Stadt Seeland Germany; ^2^ Leibniz Institute on Aging—Fritz Lipmann Institute (FLI) Jena Germany; ^3^ Plant Genome and System Biology (PGSB) Helmholtz Center Munich German Research Center for Environmental Health (GmbH) Neuherberg Germany

**Keywords:** hierarchical shotgun sequencing, bacterial artificial chromosome, sequence assembly, paired‐end sequencing, mate‐pair sequencing, physical map, *Hordeum vulgare*, barley

## Abstract

Hierarchical shotgun sequencing remains the method of choice for assembling high‐quality reference sequences of complex plant genomes. The efficient exploitation of current high‐throughput technologies and powerful computational facilities for large‐insert clone sequencing necessitates the sequencing and assembly of a large number of clones in parallel. We developed a multiplexed pipeline for shotgun sequencing and assembling individual bacterial artificial chromosomes (BACs) using the Illumina sequencing platform. We illustrate our approach by sequencing 668 barley BACs (*Hordeum vulgare* L.) in a single Illumina HiSeq 2000 lane. Using a newly designed parallelized computational pipeline, we obtained sequence assemblies of individual BACs that consist, on average, of eight sequence scaffolds and represent >98% of the genomic inserts. Our BAC assemblies are clearly superior to a whole‐genome shotgun assembly regarding contiguity, completeness and the representation of the gene space. Our methods may be employed to rapidly obtain high‐quality assemblies of a large number of clones to assemble map‐based reference sequences of plant and animal species with complex genomes by sequencing along a minimum tiling path.

## Introduction

Hierarchical shotgun sequencing is a divide‐and‐conquer strategy for genome sequencing that has been successfully applied to produce high‐quality reference genome sequences for many plant and animal species, among them *Arabidopsis thaliana*, rice and maize (Arabidopsis Genome Initiative, 2000; International Rice Genome Sequencing Project, 2005; Schnable *et al*., 2009). The main steps in hierarchical shotgun sequencing are (i) the construction of large‐insert clone libraries, (ii) building a physical map and (iii) sequencing a minimum set of overlapping clones that cover the entire genome (a minimum tiling path). Currently, large‐insert clone libraries consist in most cases of bacterial artificial chromosomes (BACs) (Shizuya *et al*., 1992). Physical maps are constructed by determining the size or direct sequencing of restriction fragments (Luo *et al*., 2003; van Oeveren *et al*., 2011). Until very recently, sequencing of minimum tiling paths has been performed using automatized Sanger sequencing (Groenen *et al*., 2012; Howe *et al*., 2013), although next‐generation sequencing (NGS) platforms have been applied for large‐scale clone sequencing (Choulet *et al*., 2014; Li *et al*., 2015; Zhang *et al*., 2012).

The main alternative to a map‐based genome sequencing strategy is whole‐genome shotgun sequencing (WGS), which is nowadays equivalent to deep sequencing of libraries of randomly fragmented genomic DNA with NGS instruments. The main advantage of a map‐based sequencing strategy over a whole‐genome approach is the reduction of genome complexity by biotechnological means prior to computational analysis, which greatly eases the algorithmic challenges associated with short‐read assembly. Sequence reads that originate from regions that are repeated hundreds or even thousands of times across the whole genome may be present at low copy number on a single BAC clone that is about 100–150 kb in size. Although the initial discussion about the relative benefits of whole‐genome and hierarchical methods was led with great intensity (Green, 1997; Weber and Myers, 1997), it is now accepted that there is no black‐and‐white distinction between both approaches.

Complementary map‐based and whole‐genome sequencing approaches are currently employed to develop genomic resources for the Triticeae. This tribe comprises several closely related cereal grasses of great agronomic importance such as bread and durum wheat, barley and rye. In spite of their economic importance, high‐quality reference genome sequences are still missing for these species, mainly owing to the large size (>5 Gb) and repeat‐rich and often polyploid structure of their genomes (Feuillet *et al*., 2012). In the last 5 years, progress in Triticeae genomics has followed three main paths: (i) shallow‐coverage whole‐genome sequencing data from flow‐sorted chromosomes and transcriptome assemblies from NGS‐data were integrated with dense genetic maps and collinearity information to construct so‐called GenomeZippers (International Wheat Genome Sequencing Consortium, 2014; Martis *et al*., 2013; Mayer *et al*., 2011); (ii) gene space assemblies were made from deep‐coverage sequencing data on the whole‐genome or whole‐chromosome level and ultra‐dense sequence‐based genetic maps (Chapman *et al*., 2015; International Barley Genome Sequencing Consortium, 2012; International Wheat Genome Sequencing Consortium, 2014; Mascher *et al*., 2013a) (iii) in parallel to the first two efforts, collaborative large‐scale physical mapping projects were initiated to construct BAC‐based physical maps of the whole genome (barley) or individual chromosome arms (wheat) (Feuillet *et al*., 2012). The genomewide physical map of barley has been completed (Ariyadasa *et al*., 2014), as has the physical map of several wheat chromosomes or chromosome arms (Breen *et al*., 2013; Lucas *et al*., 2013; Paux *et al*., 2008; Philippe *et al*., 2013; Poursarebani *et al*., 2014; Raats *et al*., 2013). Clone‐by‐clone sequencing of the entire minimum tiling path of wheat chromosome 3B has been performed using a hybrid approach combining data from the Roche 454 and Illumina NGS platforms (Choulet *et al*., 2014). Scaling BAC sequencing from a single 1‐giga‐base‐pair chromosome to the 5 Gb barley genome or the remaining twenty wheat chromosomes—each several hundred mega‐base pairs in size—will require the efficient utilization of the most advanced high‐throughput sequencing technologies.

The aim of the present study was to establish a reliable method to sequence and assemble a large number of BAC clones in a standardized manner. We aimed at assembling BACs individually, because our prior experiences with sequencing barley BACs had indicated that joint assembly of reads from multiple clones results in the formation of chimeric sequence contigs (Taudien *et al*., 2011). Thus, sequencing libraries should be constructed for individual BAC clones to track the origin of each sequence read and enable the assembly of reads from only single BACs. Consequently, pooling should be performed only after construction of barcoded libraries as opposed to sequencing pools of BACs without barcoding and then (i) assembling a larger genomic segment formed by overlapping BACs, or (ii) pooling nonoverlapping BACs and relying on the assembly algorithms to separate reads from unrelated regions.

Here, an integrated wet laboratory and computational pipeline for sequencing, assembling and scaffolding individually barcoded BAC clones is presented. The pipeline was employed to sequence BACs from the minimum tiling path of the barley genome.

## Results

### Overview of the pipeline

**Figure 1:**
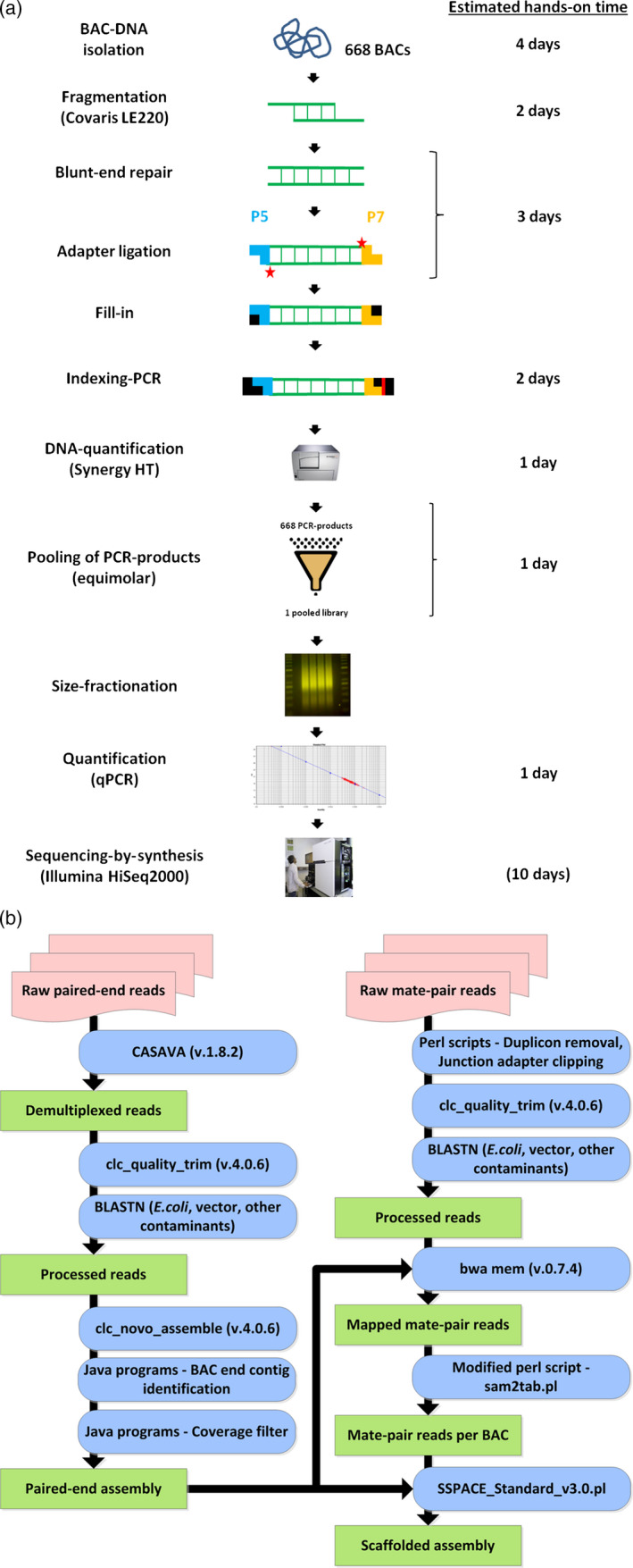
Overview of the BAC sequencing and assembly pipeline. (a) Wet laboratory workflow. The estimated hands‐on times of each step are shown in the right column of panel A and indicate the amount of time each step may require if two technicians process about 650–700 BACs (96‐well plate format). The number in brackets in the last row refers to the operating time of the Illumina HiSeq 2000 device when performing 2 × 100 cycles for paired‐end sequencing. (b) Assembly pipeline. Input data (red boxes) are subjected to several processing steps (blue boxes), producing respective output (green boxes). The programs and scripts used in each step are indicated within the boxes.

In the wet laboratory part of our pipeline, multiplexed Illumina sequencing libraries are constructed from DNA of individual BAC clones (Figure 1a). After sequencing, resultant read data are automatically processed by the computational part of the pipeline (Figure 1b). Parallelization is employed to speed up the assembly process. The source code of our pipeline is available for download at http://dx.doi.org/10.5447/IPK/2015/6. In the following, we give a detailed walkthrough of our pipeline by following the sequencing and assembly of 668 BACs, whose inserts mostly originate from barley chromosome 4H.

### Shotgun sequencing of barley BACs and quality control

The set of 668 clones included 653 BACs that originated from barley. These BACs contain clones from two of the six independently constructed BAC libraries (Schulte *et al*., 2011) that have been previously designated as part of the minimum tiling path of the barley genome by restriction fragment‐based fingerprinting and subsequent clone overlap analysis (Ariyadasa *et al*., 2014). Moreover, we chose fifteen ‘reference BACs’ to be used as internal controls for monitoring the performance of the sequencing and assembly process. These ‘reference BACs’ included two human BAC clones (one with six; NCBI accession AF252830; and the other with eight replicates; NCBI accession AF285443) as well as one barley BAC clone that had already been shotgun‐sequenced repeatedly (NCBI accession AY268139) (Steuernagel *et al*., 2009; Taudien *et al*., 2011). The manually curated assemblies of each of the ‘reference BACs’ consisted of a single sequence contig and are available from NCBI (Table S2).

**Figure 2:**
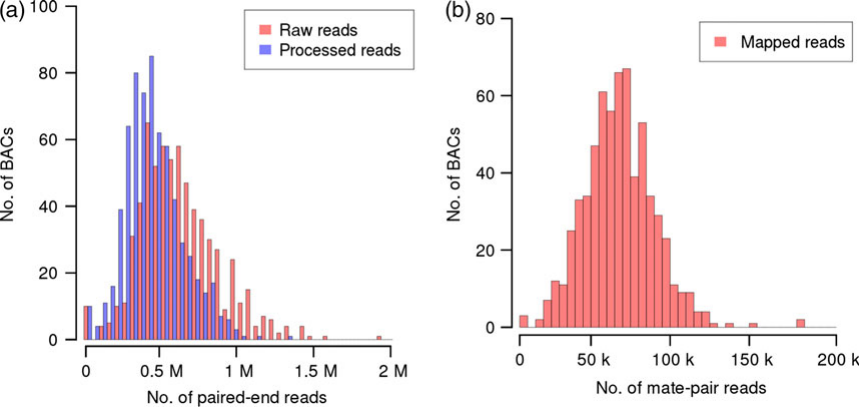
Sequencing statistics. (a) Histograms of the number of raw (red) and processed (blue) reads assigned to each BAC. Processed reads were used as input for *de novo* assembly. (b) Distribution of the number of mate‐pair reads assigned to each BAC. Mate pairs were mapped to the initial paired‐end BAC assemblies. Only uniquely mapping pairs were assigned to BACs.

To sequence hundreds of BACs in one lane of the Illumina HiSeq 2000 sequencing device, we adopted a versatile protocol for deep multiplexing of templates (Meyer and Kircher, 2010) that we had previously used for genotyping by sequencing (Wendler *et al*., 2014). Briefly, the BAC DNA was fragmented and ligated to sequencing adaptors to serve as templates for index PCR, yielding 668 individually barcoded BAC libraries. Full details of the protocols used for library preparation and sequencing are described in the method section. All 668 individual libraries were subsequently pooled in an equimolar manner and sequenced in a single lane of the Illumina HiSeq 2000 instrument using paired‐end chemistry (2 × 100 cycles). After deconvolution of 418 million raw reads (42 Gb of sequence, Table 1), an average of 625 170 reads could be assigned to each individual BAC (minimum: 308, maximum: 1 933 970, Figure 2a). Assuming an average genomic insert size of 145 kb, the mean read depth was 431. Reads originating from BAC vector backbone, the *E. coli* genome or reads of low‐quality (12% of reads were below Q30) were excluded from further analysis. This process removed an average of 29% of raw reads per BAC. After filtering, the average read depth per BAC was 307 (Table 1).

**Table 1:** Sequencing output for 668 BACs (paired‐end and mate‐pair sequencing)

Library	Insert size	Raw read length	Processed length of single reads	No. of reads (raw)	No. of bases (raw)	No. of reads (processed)	No. of bases (processed)	Duplication rate^a^, %
Paired‐end HiSeq	500 bp	2 × 100 bp	97 bp	417 613 642	41 970 171 021	297 035 172	28 897 856 622	0.43
Mate‐pair MiSeq library1	5500 bp	2 × 250 bp	152 bp	5 270 554	855 965 539	2 829 040	429 345 869	0.69
Mate‐pair MiSeq library 2	5700 bp	2 × 250 bp	147 bp	5 085 586	799 162 083	2 754 524	405 327 532	0.85
Mate‐pair HiSeq library 1	5900 bp	2 × 100 bp	82 bp	34 897 774	3 472 328 513	17 171 286	1 409 064 253	3.61
Mate‐pair HiSeq library 1	6200 bp	2 × 100 bp	82 bp	48 555 264	4 831 248 768	24 108 068	1 966 403 705	5.59

a Proportion of duplicated reads. These arose either during PCR reactions or during sequencing‐by‐synthesis [optical duplicates, see Whiteford *et al*. (2009)].

### *De novo* assembly of paired‐end data

**Figure 3:**
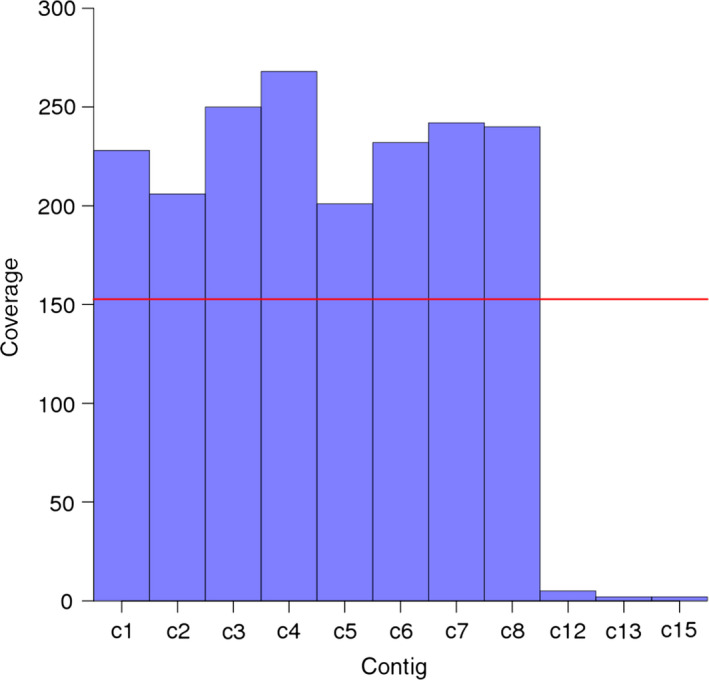
Motivation for implementing a coverage threshold to exclude contigs with low read depth. The average read depth is shown for all contigs of the initial paired‐end assembly of BAC clone HVVMRXALLeA0276J07. Contigs with coverage below 153 were removed from the assemblies. This threshold was calculated by the algorithm as 50% of the average contig coverage. BLAST searches revealed that the excluded contigs (c12, c13 and c15) originated from the bacteriophage phiX genome, which is included as an internal quality control for sequencing (recommended by Illumina as a low concentration spike in). Note that contigs c9, c10, c11 and c14 had been removed because they were entirely composed of sequence originating from the vector or the *E. coli* genome.

We performed initial sequence assemblies separately for each BAC with CLC Assembly Cell software using all filtered paired‐end reads. We note that the CLC assembler includes a scaffolding step attempting to link contigs using paired‐end information. In the following, we will refer to the initial CLC scaffolds as contigs because no long‐distance mate‐pair data were used by CLC. The initial set of sequence contigs was further processed by removing remaining vector ends. Flexible coverage thresholds were also set up to remove contigs that were supported by only a very small number of reads and most likely originated from cross‐contamination (from BACs in neighbouring wells during library preparation) (Figure 3) or due to errors during computational demultiplexing (wrongly assigned reads) (Kircher *et al*., 2012). We removed from the assembly of each BAC those contigs that had a coverage below half the mean coverage calculated across the set of contigs after removing 10% of contigs with the lowest coverage. This step purged the final assemblies of contigs that resulted from reads originating from other BACs and were present with low frequency in the deconvoluted reads. From an initial number of 16 313 contigs, 8282 (50.8%) were removed. The discarded sequence set of contigs was mainly characterized by small sequence length. The average length of low coverage contigs was 1600 bp as compared to 10 208 bp for all contigs. Thus, the cumulative low coverage contig length was 13 128 kb and accounted for 12% of the cumulative raw assembly length.

**Figure 4:**
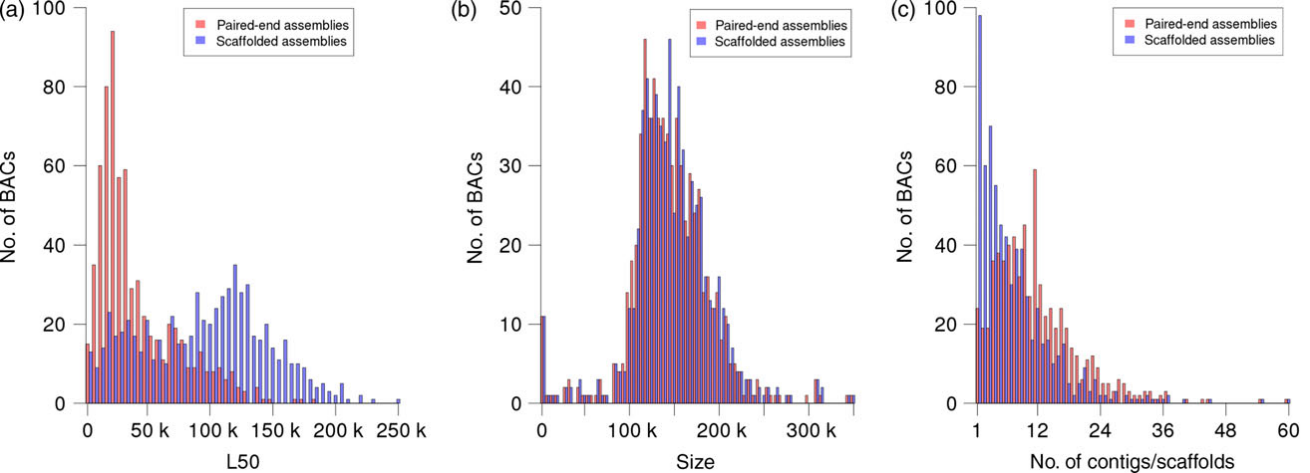
Assembly statistics. The three panels show the distributions of L50 values (a), assembly sizes (b) and the numbers of contigs (red) or scaffolds (blue) (c) of all individual BAC assemblies. Histograms for the initial paired‐end assemblies are shown in red, those of assemblies after scaffolding with mate‐pair reads are shown in blue. L50 is defined as the length of the smallest contig (or scaffold) among all size‐ranked (from longest to shortest) contigs (or scaffolds) that together make up 50% or more of the length of a given assembly. The assembly size is the sum of the lengths of all constituent contigs or scaffolds.

After postprocessing, BAC assemblies consisted of an average of twelve contigs (Table 2, Figure 4c). The mean combined length of all contigs per BAC was 146 kb, with a range between 827 bp and 345 kb (Figure 4b). On average, half of the sequence of each BAC was contained in contigs larger than 42.3 kb, which is similar to our previous assembly results with Roche 454 read data (Figure 4a) (Steuernagel *et al*., 2009; Taudien *et al*., 2011).

**Table 2:** Assembly summary statistics^a^

	Before scaffolding	After scaffolding
Number of contigs/scaffolds per BAC	12.2	7.8
L50^b^	41 346 bp	94 887 bp
Size of largest contig/scaffold	55 072 bp	100 261 bp
Assembly length	146 436 bp	147 830 bp
Assembly length in contigs/scaffolds ≥1 kb	145 135 bp	146 555 bp
Assembly length in contigs/scaffolds ≥10 kb	116 391 bp	133 281 bp

a Assembly statistics were calculated for each BAC. Then, the arithmetic mean across all 668 values was computed.

b The L50 is maximum value L such that 50% of the assembly length is contained in contigs whose size is greater than or equal to L.

### Scaffolding with Nextera mate‐pair reads

Sequencing projects have to employ several library types and computational tools to improve the contiguity and completeness of sequence assemblies. Scaffolding refers to improving an initial sequence assembly that is fragmented into multiple contigs using long‐distance mate‐pair reads to connect contig sequences and bridge gaps in the assembly. This process also involves ordering and orienting the set of contigs relative to each other and estimating the sizes of gaps between them. We sequenced 8 kb Nextera mate‐pair libraries of BAC pools to improve the contiguity of the initial BAC assemblies made only from short‐insert paired‐end reads. For preparing mate‐pair libraries, the 668 BAC clones were distributed into two nonoverlapping subpools each containing 334 BACs. Both libraries were sequenced twice (Table 1): once in one‐tenth of a single‐lane flow cell of the Illumina MiSeq, yielding 1.6 Gb of sequence (2 × 250 nt reads), and once in one‐eleventh of one lane of the Illumina HiSeq 2000, yielding 8.3 Gb of sequence (2 × 100 nt reads). Sequencing the libraries on two different platforms combined the advantages of higher read length of the MiSeq instrument with the larger sequencing output of the HiSeq 2000 device.

**Figure 5:**
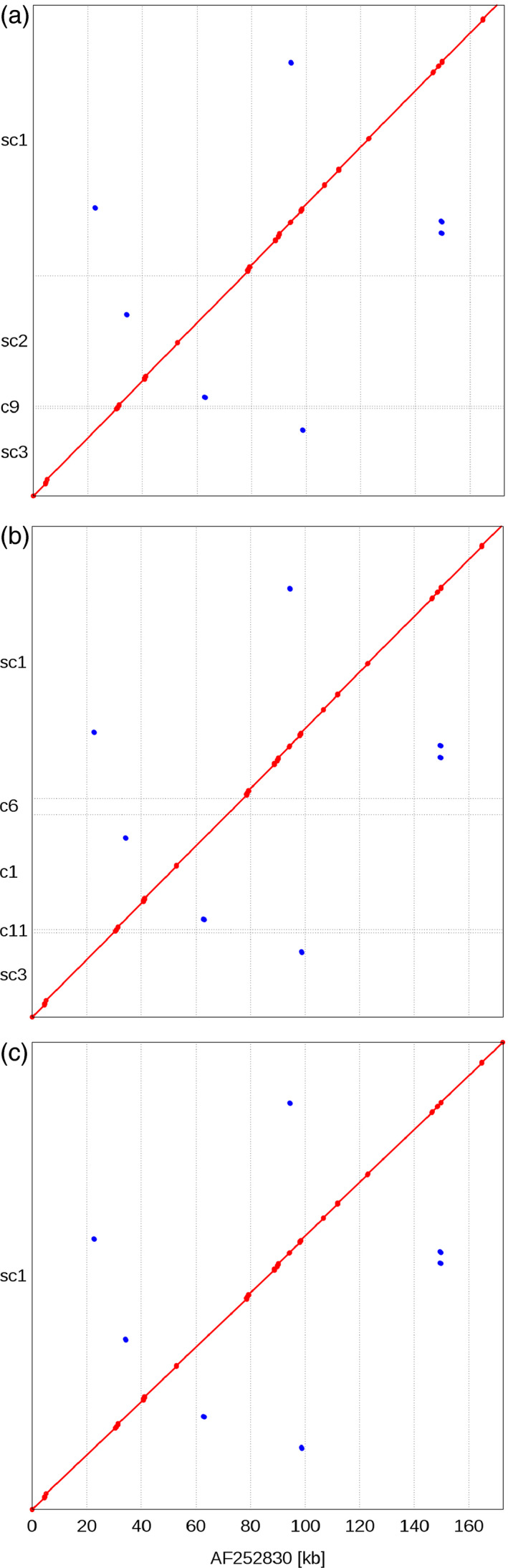
Alignment of human control BAC assemblies against their finished reference assembly. Three assemblies of one human reference BAC clone from independent sequencing libraries were aligned to the manually curated reference assembly obtained from Sanger sequencing data (available as NCBI accession AF252830). Red diagonals indicate sequence identity of the alignment coordinates between each of our three *de novo* and the public reference assembly (a–c). Red dots correspond to small gaps in the alignment that are caused by stretches of unknown bases (‘Ns’). All assemblies represent between 99% and 99.8% of the entire length of the reference assembly. Scaffold sc2 in (a) and contig c1 (b) contain a misassembled region, which may have prevented correct scaffolding with mate‐pair reads. Among the three *de novo* assemblies only the one shown in (c) consisted of a single scaffold.

No individual barcoding of BACs was applied during library preparation. Instead, read pairs were deconvoluted by mapping them to the entire set of initial paired‐end assemblies. Pairs mapping uniquely to a single BAC assembly were assigned to this BAC. 59% of MiSeq and 65% of HiSeq reads were assigned to their BAC of origin (Figure 2b). From this set of deconvoluted reads, only those pairs, whose two ends mapped to different contigs of the same BAC, were informative for scaffolding. Thus, only a minor fraction of read pairs (6.4% of MiSeq and 0.5% of HiSeq mate pairs, Table S3) were used as input for the scaffolding process utilizing the SSPACE software (Boetzer *et al*., 2011). Before scaffolding, BAC assemblies were fragmented on average into 12 contigs; after scaffolding, BAC assemblies consisted of an average of eight scaffolds. Scaffolding increased the assembly L50 on average by 53.1 kb. The mean size difference between the longest scaffold and the longest contig before scaffolding was 45.0 kb (Figure S1). Thirteen per cent of BACs (88 BACs) were represented by a single sequence scaffold. Half of the assembly length was contained in scaffolds larger than 95 kb (Table 2, information for individual BAC assemblies is available in Table S4). As scaffolding only orders and orients existing contigs, but does not introduce new sequence information—stretches of unknown bases (‘Ns’) are introduced as gaps between linked contigs—the sequence information content remains constant. Manual inspection of sequence alignments supported the good concordance of our final assemblies of the reference BACs and their “gold‐standard” assemblies in the public sequence archives (Figure 5).

### Evaluation of assembly quality

In addition to manual inspection of dot plots, we examined the quality of our BAC assemblies with computational tools specifically designed for this purpose. Inconsistencies between our BAC assemblies and their finished reference assemblies were catalogued with QUAST software (Gurevich *et al*., 2013). QUAST detects larger structural differences (misassemblies) and smaller insertions and deletions <50 bp (indels). According to the criteria defined by the Plantagora project (Barthelson *et al*., 2011), misassemblies were categorized into ‘global misassemblies’ and ‘local misassemblies’ (see Experimental procedures for full details). On average, 98.5% of reference sequence was covered by contigs of the initial paired‐end assemblies. The mean number of global and local misassemblies per BACs were 1 and 17, respectively (Table S5). Scaffolding introduced few errors: the average number of misassemblies increased to 2 (global) and 20 (local) (Table 3). By contrast, the contiguity was greatly improved: the average L50 of the fifteen reference BACs increased from 27 kb to 99 kb. In summary, scaffolding with 8 kb mate‐pair reads boosted the contiguity of the assembly without compromising its quality.

**Table 3:** Assembly quality statistics for sequenced reference BACs (scaffolded assemblies)

BAC	L50	No. of misassemblies	No. of local misassemblies	Indels	Genome fraction^a^	No. of scaffolds
NCBI_AF252830_25	97 756	2	19	10	99.8	4
NCBI_AF252830_26	99 400	1	28	2	99.0	5
NCBI_AF252830_27	179 959	0	24	9	99.3	1
NCBI_AF252830_28	76 048	1	26	10	98.8	3
NCBI_AF252830_29	97 573	1	25	12	99.3	6
NCBI_AF252830_30	69 370	1	18	15	99.0	4
NCBI_AF285443_23	39 507	1	23	6	99.2	5
NCBI_AF285443_24	97 772	2	11	8	97.2	4
NCBI_AF285443_25	146 380	3	22	11	99.2	2
NCBI_AF285443_26	47 846	2	21	7	98.7	4
NCBI_AF285443_27	147 003	1	15	33	96.2	3
NCBI_AF285443_28	43 512	4	19	18	98.0	6
NCBI_AF285443_29	150 689	0	13	9	99.6	1
NCBI_AF285443_30	85 073	3	22	11	99.5	3
HVVMRXALLhA0184G09	110 185	1	9	5	97.1	3
Mean	99 205	2	20	11	98.7	4

a Fraction of the genomic insert that is represented in the assembly.

### Representation of genes

**Figure 6:**
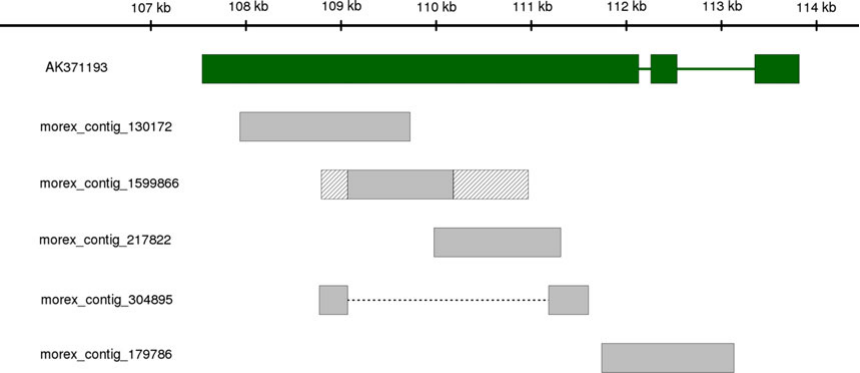
Example of a full‐length cDNA with incomplete sequence representation in the draft WGS assembly of barley. The entire sequence of full‐length cDNA clone NIASHv2127M20 (NCBI accession AK371193) can be aligned to scaffold sc1 of BAC HVVMRXALLeA0226A12 (alignment coordinates: 107 539 bp to 113 818 bp). The aligned regions are represented as green rectangles. Introns that had been spliced from the original mRNA are shown as green lines. No single WGS contig contains the entire sequence of AK371193. Parts of it are spread across five different WGS contigs, whose names are indicated on the left. Four of these contigs are completely contained in HVVMRXALLeA0226A12, although one of them (morex_contig_304895) is misassembled and joins two distant sequences (indicated by a dashed line). Another contig (morex_contig_1599866) can be aligned only partly to HVVMRXALLeA0226A12. The sequences at its end (indicated by the hatched area) are misassembled sequences. Paralogous, possibly pseudogenized, copies of the gene may have complicated assembly from whole‐genome sequencing data.

We assessed whether the higher contiguity of our BAC assemblies (as compared to published draft WGS assembly) improved the representation of genes for which no or only partial sequence information was available in the WGS assembly. In total, 26 159 high‐confidence gene models have been predicted for barley (International Barley Genome Sequencing Consortium, 2012). We searched barley full‐length cDNAs (Matsumoto *et al*., 2011) for coding sequences that were (almost) completely contained in a single sequence scaffold of the BAC assemblies, but spread across multiple WGS contigs or partially absent from the WGS assembly. We found twelve such examples, one of which is shown in Figure 6. The cumulative length of all unique barley BACs assembled in the present study is 95 Mb. Extrapolating from the twelve full‐length cDNAs that we found to be represented better in the BAC assemblies, we can estimate that a minimum of five hundred gene models may be fragmented or incomplete in the WGS assembly. This is only a very rough estimate that does not take into account differences in gene density along the genome. Genotypic differences can be excluded as the explanation for ‘missing’ genes as both the BAC libraries and the WGS assembly were derived from cultivar Morex. To conclude, the construction of a BAC‐based reference sequence can be expected to improve the sequence representation of the barley gene space.

### Repetitive sequences

**Figure 7:**
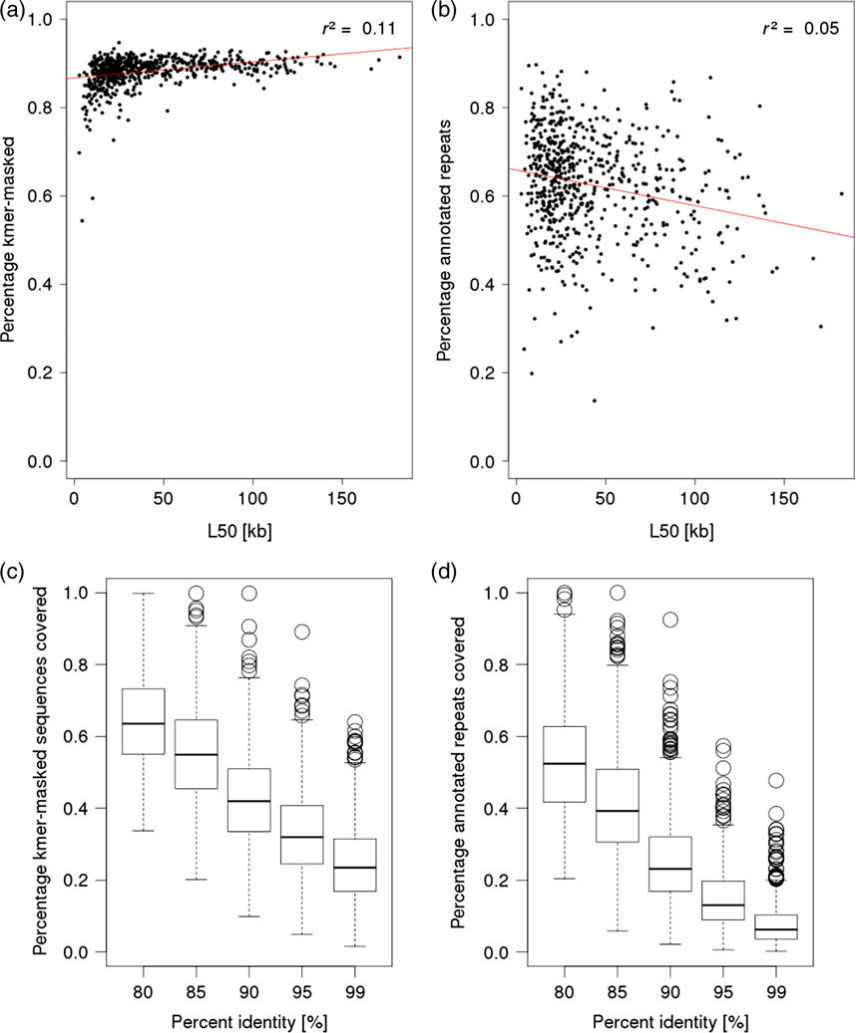
Analysis of repetitive sequence content. The panels a and b show the relation between the amount of repetitive sequences and the L50 of the barley BAC assemblies. In both panels, the red line shows the linear regression curve for these values determined with R command ‘lm’. The r^2^ values are indicated in the plots. The box plots in panels c and d show the distribution of fraction of repetitive sequences in the BAC assemblies matching to WGS for different alignment identity thresholds (80–99%). Repetitive sequences were identified using a *k*‐mer‐based approach in panels a and c and a search against known repeat families in panels b and d.

We wished to assess whether the repeat content of single BAC is correlated with final assembly contiguity. Towards this aims, we applied the Kmasker software to identify stretches of highly abundant 21*‐*mers in the assemblies using the reference index for *k*‐mers occurring in the barley genome (Schmutzer *et al*., 2014). On average, 88.2% of the BAC assembly sequences were classified as being composed of 21‐mers that are highly abundant in the barley genome (Figure 7a). Screening the BAC sequence scaffolds for the presence of known repeat families from the Triticeae Repeat Sequence Database (Wicker *et al*., 2002) assigned on average 62.5% of the total sequence of the BAC assemblies to annotated repeats (Figure 7b). There was no clear correlation between the repeat content of BACs with the contiguity of their sequence assemblies as measured by the L50 value, indicating that overall repeat content is not a good predictor of final BAC assembly quality (Figure 7a and b).

Next, we tested whether the representation of repetitive sequences is better in the BAC assemblies than in the WGS assembly of cv. Morex. Aligning the WGS contigs to repeat structures identified by either the Kmasker approach, or to annotated repeats found in the BAC assemblies showed that 24.8% of *k*‐mer masked sequences and 7.9% of annotated repeat sequences were covered by WGS contigs with a minimal alignment identity of 99%. At a lowered identity threshold of 80%, however, the majority of repeats had matches to WGS assembly (Figure 7c and d), indicating that highly similar copies of repetitive elements were often collapsed into ‘consensus contigs’ in the WGS assembly, but were better resolved in the single BAC assemblies.

## Discussion

We have established a high‐throughput pipeline for sequencing and assembling a large number of BAC clones. The two main improvements of our pipeline over previous efforts for BAC sequencing are (i) deep multiplexing of samples on the Illumina HiSeq 2000 platform (ii) and use of updated bioinformatics protocols to accommodate the new structure and vastly increased amount of raw data. Previous BAC sequencing protocols were set up for the Roche 454 sequencing platform (Choulet *et al*., 2010; Steuernagel *et al*., 2009; Taudien *et al*., 2011) or for sequencing smaller numbers of clones on the Illumina platform using standard multiplexing procedures (Sathuvalli and Mehlenbacher, 2013).

The aim of BAC sequencing can be stated easily, to represent the entire nucleotide sequence of the genomic insert by a single sequence contig. In the present study, this task was complicated by great abundance and high copy of transposable elements in the barley genome, which prevented assembling BACs into a single contig in most cases. Although the assemblies represented on average >98% of the inserts, they remain fragmented into multiple contigs with an L50 of 95 kb. This is not unexpected. The most important precedent for assembling a large and repeat‐rich genome is the human genome project. When the first working draft of the human genome was published, ~ 30 000 large‐insert clones were sequenced with Sanger sequencing technology and were represented by 400 000 contigs with an L50 of 83 kb (Kent and Haussler, 2001). The final assembly of the BACs into single contigs required labour‐intensive manual curation (International Human Genome Sequencing Consortium, 2004). We anticipate that further improvements to the contiguity and genome representation of BACs comprising the minimum tiling path of the barley genome will proceed along the following lines: (i) generation and use of additional genomewide data sets to improve the contiguity of clone assemblies; (ii) use of long‐read sequencing platforms to close remaining gaps (English *et al*., 2012). Additional genomewide data sets may include deeply sequenced mate‐pair libraries of various insert size, chromatin conformation capture sequencing (HiC) data (Burton *et al*., 2013; Kaplan and Dekker, 2013) or *in vitro*‐derived long‐range connectivity information (Adey *et al*., 2014). The data sets required for these steps cannot be collected at the level of single clones or larger BAC pools because the costs associated with doing so would be prohibitively high, or because working with BACs, as in the case of chromatin conformation capture sequencing strategies, may be meaningless. To utilize these genome‐scale NGS‐based techniques for scaffolding and gap‐closing, a genomewide, nonredundant sequence assembly is necessary. Therefore, a pipeline to automatically detect and visualize overlaps between clones was set up (Colmsee *et al*., 2015). This sequence overlap information will be used to construct nonredundant sequences on the level of finger‐printed contigs, where adjacent clones overlap by approximately 30 kb. It is important to note that 90% of the assembled sequence of our BACs is contained in contigs ≥10 kb, which allows to retrieve long (>10 kb), high‐identity (>99.5%) overlaps between adjacent BACs. These overlaps can be well distinguished from regions of high sequence identity shared between copies of transposable elements on unrelated BACs. Thus, the immediate next goal of the International Barley Genome Sequencing Consortium is to finish the sequencing of all 66 772 clones of the minimum tiling path of the barley genome (Ariyadasa *et al*., 2014) and to construct an initial nonredundant clone‐based sequence assembly of the entire barley genome.

New sequencing technologies involving single‐molecule sequencing (Eid *et al*., 2009) or nanopore devices (Goodwin *et al*., 2015) hold the potential of producing BAC assemblies of improved contiguity. Current single‐molecule sequencing as marketed by Pacific BioSciences Inc. (PacBio) has been shown to deliver high‐quality assemblies of entire bacterial genomes (Koren and Phillippy, 2015). Thus, PacBio sequencing can be expected to yield finished single‐contig assemblies of BACs using only automated procedures. PacBio continuously improves the performance of its sequencing devices and has also introduced protocols for multiplex sequencing. Based on currently published results, however, it is not clear to us whether the cost and throughput of the PacBio sequencing platform will be sufficient for sequencing a large number of BAC clones. Moreover, sequencing entire minimum tiling paths may require the development of new protocols for deep multiplexing protocols for the PacBio platform. Given the progress in the development of algorithms that can efficiently employ long, albeit error‐prone reads for the assembly of eukaryotic genomes (Kim *et al*., 2014; Koren and Phillippy, 2015), it can be envisaged that a whole‐genome shotgun approach combined with single‐molecule sequencing can be used to assemble huge plant genomes in the future.

Current standard protocols allow an academic laboratory to manually generate thousands of barcoded libraries from individual BAC DNAs for sequencing without major investments, except the Covaris LE220 instrument required for the fragmentation of DNA. To increase the throughput of library construction further and to reduce the manual labour time, the use of precision liquid handling robots might be beneficial. The standard protocol can be adapted readily to automation. Library preparation is already parallelized right from the start in the 96‐well format and the enzymatic reactions mostly involve pipetting volumes of at least 20 μL, which are suitable for automated processing. Only the set‐up of the index PCR might require some minor alterations to increase the volumes of index primer and template DNA. Reaction clean‐up steps are based on paramagnetic beads (SPRI method) and are amenable for automation as well. If the set‐up of a fully automated robot system is impractical, manual easy‐to‐use 96‐well pipetting systems (PlateMaster; Gilson International) are an interesting additional option to accelerate liquid handling. An alternative to the final manual gel‐based library purification is the use of the automated BluePippin device (sage science) or double‐SPRI size selection (Lennon *et al*., 2010; Rodrigue *et al*., 2010). All these alternatives might be worthwhile additions to further reduce costs or increase throughput of single BAC sequencing.

Development and application of the BAC sequencing pipeline described in this manuscript were motivated by our involvement in the barley genome sequencing project (Schulte *et al*., 2009). Obviously, the construction of BAC libraries, physical mapping and clone sequencing are not limited to plant genomics. However, map‐based genome sequencing is of particular interest to plant research, because of the complex structure of many plant genomes (large genomes >5 Gb and abound in repetitive elements), which is not amenable for whole‐genome shotgun approaches. For instance, WGS assemblies of barley (5 Gb), white spruce (20 Gb) and hexaploid bread wheat (17 Gb) achieved only 40–70% genome representation. The missing sequence presumably originates predominantly from families of repetitive elements whose individual copies are highly similar to each other and are thus prone to collapse into a single contig in the assembly. Obtaining assemblies that correctly represent repetitive regions is an important goal of reference genome projects in repeat‐rich plant genomes. Transposable elements act as driving forces of genome evolution (Fedoroff, 2012), and furthermore, they are involved in (i) the diversification of centromeres (Gao *et al*., 2015), (ii) the rapid adaptation to changing environments (Stapley *et al*., 2015) and (iii) the expression of genes near their insertion sites (Makarevitch *et al*., 2015). In addition the benefits of a map‐based reference sequence are not limited to a better resolution of repeat regions, but also improve the representation of genic regions. In the present study, we have shown that in contrast to the barley WGS assembly the sequence of full‐length cDNAs is faithfully represented in BAC assemblies. The apparent absence of *bona fide* genes from the WGS assembly of barley reinforces the conclusion of Denton *et al*. (2014) that inferences on the number of genes and the sizes of gene families from draft genome assemblies should be treated with caution.

Currently the pipeline described in the present paper is used to sequence and assemble the complete minimum tiling path of the barley genome. Our methods can be readily extended to wheat genome sequencing as all relevant resources—BAC libraries, physical maps and supporting whole‐genome data sets—are in place or are being generated. Moreover, we anticipate that our pipeline will be valuable for other sequencing project in species with large genomes.

## Experimental procedures

### Preparation of shotgun sequencing libraries

The isolation of BAC DNA was based on the alkaline lysis method (Birnboim and Doly, 1979). Shotgun sequencing of up to 668 individually barcoded BACs (Illumina multiplex library) was performed essentially as described previously (Meyer and Kircher, 2010). Briefly, BAC DNA was fragmented using focused acoustic energy (Covaris E220) and cleaned up by precipitation using SPRI‐bead suspension (Himmelbach *et al*., 2014). The DNA was eluted and blunt‐end repaired as described (Himmelbach *et al*., 2014). The fragments were provided with two adapters (P5, P7) and SPRI purified (Himmelbach *et al*., 2014). The adapter ligated DNA was filled in using *Bst* DNA polymerase (large fragment), SPRI purified and processed for indexing PCR (Himmelbach *et al*., 2014). 672 different index primers (Table S6) were created according to the previously published guidelines (Meyer and Kircher, 2010). The PCR products were pooled in equimolar manner and size‐selected (420–520 bp) using agarose gel electrophoresis (Himmelbach *et al*., 2014). The structure of the adaptor‐ligated DNA fragments is provided as Figure S2. Libraries were quantified using real‐time PCR (Mascher *et al*., 2013a, b). Cluster formation (cBot), 2 × 100 cycles paired‐end sequencing and index read (8 cycles) were performed according to the manufacturer (Illumina, San Diego, California, USA) instructions. Sequences were extracted by CASAVA version 1.8.2 (GenomeAnalysis‐Pipeline).

### Preparation of Nextera mate‐pair sequencing libraries

Nextera libraries were prepared following essentially the instructions of the manufacturer (Illumina, FC‐132‐1001). Libraries were quantified and sequenced as described for shotgun sequencing libraries. Up to 12 libraries (each constructed from 384 BACs) were sequenced in one HiSeq 2000 lane (2 × 100 cycles). In addition, up to six libraries were sequenced using the Illumina MiSeq (2 × 250 cycles). FASTQ sequence files were extracted with CASAVA version 1.8.2 (HiSeq2000) or with the MiSeqReporter Software (Illumina, San Diego, California, USA). Full details of all wet laboratory procedures are described in Data S1.

### Initial assembly of paired‐end reads

Sequence assembly of data obtained for large BAC pools was performed with a customized pipeline for preprocessing, assembly and postprocessing. The source code of all scripts is available for download using the digital object identifier (DOI) http://dx.doi.org/10.5447/IPK/2015/6. This DOI was created with e!DAL (Arend *et al*., 2014). The pipeline consists of two major modules, one written in Java 1.6 that handles thread initialization and execution while the second module written in Perl 5.8 handles the *de novo* assembly of individual BAC clones. Users can specify the number of parallel assemblies to be run by the pipeline. Internally, standard Java functions (ThreadPoolExecuter, BlockingQueue) ascertain that the number of active threads does not exceed the number of available CPU cores. Each individual BAC was allocated a single execution thread, in which assembly with CLC and BLAST searches during contamination removal steps were run consecutively. This parallelization layout is based on the ThreadPoolExecuter implementation in Java and works in conjunction with a queue of all tasks (i.e. the number of all FASTQ files in the assembly input folder).

### Pre‐assembly processing and quality control

To address the problem of previously reported poor read quality at the 3′ ends of Illumina sequenced reads (Minoche *et al*., 2011) clc_quality_trim (version 4.0.6 beta) was used on every BAC read set individually with default parameters and only keeping pairs. Genomic *E. coli* reads were removed through BLASTN filtering with an e‐value cut‐off of 1 × 10^−5^. Vector reads were partially kept for initial assembly if originating up to 2 kb up or downstream of the cloning site [adapted from (Steuernagel *et al*., 2009)], the remainder of vector reads was discarded (BLASTN with an e‐value cut‐off of 1 × 10^−5^).

### Postprocessing of assemblies

Assemblies were performed by CLC Assembly Cell (version 4.0.6 beta; CLCbio, Aarhus, Midtjylland, Denmark) using the command clc_novo_assemble. Single BAC assemblies were performed using 10 CPUs (parameter –cpus 10). Insert size parameters were set to ‘‐p fb ss 200 1000’ according to the span sizes of paired‐end libraries. The resultant contigs were then screened for the remaining vector sequences using cross‐match. Vector sequences almost exclusively assembled to the ends of a contig making it possible to mark these as ‘BAC end contigs’. After clipping vector sequences, only contigs with a length of at least 500 bp were kept. To account for possible contamination either between BAC clones or other sources, reads were mapped against the clipped version of the assembly by running megaBLAST (e‐value cut‐off of 1 × 10^−5^). The best hit of each read was regarded as position assigned by the assembler during contig production. Furthermore, the total length of all reads from a single contig was summed and then divided by the contig length to deduce contig read coverage. Furthermore, the average contig coverage of all contigs from an individual BAC clone was calculated and a filter threshold of 90% of half of the average coverage was set [to account for uneven coverage in the sequenced region (Dohm *et al*., 2008)]. Contigs with a read coverage lower than this threshold were marked as possible contamination or assembly artefact and removed from the assembly.

Finally, assemblies were screened against common contaminants (including sequencing adaptors, common phage sequences, common bacterial sequences, vector sequences; DOI: http://dx.doi.org/10.5447/IPK/2015/6) with megaBLAST (Zhang *et al*., 2000) using a minimum sequence identity of 90% and a minimum alignment length of 100 bp. Detected contaminants were removed from the assembly by either clipping contig ends or completely removing the contaminated sequence if the contig length after clipping was below 500 bp.

### Preprocessing and deconvolution of mate‐pair reads

Mate‐pair reads were processed using a custom PERL script for identifying and discarding duplicate read pairs. The read pairs were screened for the Nextera junction adapter and clipped according to the outline in the Illumina guideline (http://www.illumina.com/documents/products/technotes/technote_nextera_matepair_data_processing.pdf) using BLASTN (Altschul *et al*., 1990) with an e‐value cut‐off of 1 × 10^−5^. Reads were clipped according to the orientation of the read and location of the adapter in the read following the procedures described by Leggett *et al*. (2014). After clipping, only reads with a length >15 bp were kept. Unpaired reads were discarded.

Quality trimming was then performed to remove poor quality bases with clc_quality_trim (version 4.0.6 beta) with default parameters. Reads containing vector or *E. coli* sequences were removed using BLASTN with an e‐value cut‐off of 1 × 10^−5^.

The draft assemblies of all 658 BACs were concatenated into a single file and used as reference to deconvolute the mate‐pair reads in the following fashion. BWA‐MEM version 0.7.4 (Li and Durbin, 2009) was used to map the preprocessed mate‐pair reads against the paired‐end draft assemblies. Estimated span sizes of read pairs were obtained by filtering reads for mapping quality by SAMtools version 0.1.18 (Li *et al*., 2009) and then calculated mean insert sizes by a custom script filtering the mapped data set for high quality (using the command ‘samtools view ‐q 40’). Only read pairs mapped to the same contig were considered for insert size estimation. A modified custom PERL script based on the SSPACE program sam2tab.pl was then used to assign read pairs to individual BAC assemblies.

### Iterative scaffolding

MiSeq and HiSeq 2000 libraries from the same pool were regarded as two different libraries in scaffolding to prevent potential issues caused by large differences in sequence coverage between the two libraries. The MiSeq library was processed first. Scaffolding was performed with SSPACE_Standard_v3.0 (Boetzer *et al*., 2011). The value of the SSPACE parameter –k (minimal number of links between contigs) was set to ten for MiSeq and twenty for HiSeq mate pairs. The standard deviation of the insert size was set to 50% of the mean. Each BAC was scaffolded independently. After scaffolding with the MiSeq data, assemblies were concatenated and used as reference for deconvoluting the HiSeq mate‐pair read data. Scaffolding with the HiSeq mate‐pair reads was performed identical as described for the MiSeq data.

### Sequence comparison of reference BACs

The programs ‘nucmer’ and ‘mummerplot’ from the MUMmer package version 3.23 (Kurtz *et al*., 2004) were used to align the assemblies (prior and after scaffolding) of the reference BACs to their sequences in the public archives (Tables S5 and 3). Assembly quality statistics were obtained with QUAST version 2.3 (Gurevich *et al*., 2013) using default parameters). Misassemblies were classified according to Plantagora (Barthelson *et al*., 2011). Briefly, a ‘misassembly’ is characterized as a position in the contigs where either: (i) the left and the right flanking sequences align over 1 kb away from each other, (ii) the flanking sequences overlap for more than 1 kb, or (iii) the flanking sequences align to different strands. A ‘local misassembly’ is a position that has to fulfil the following three conditions: (i) there are two or more distinct alignments that cover the position, (ii) its flanking sequences align less than 1 kb apart and (iii) both flanking sequences align to the same strand. Misassemblies that do not qualify as local misassemblies are considered as ‘global misassemblies’. For visualization, dot plots were produced using the MUMmer program suite (Kurtz *et al*., 2004).

### Analysis of repeat content

*K*‐mer frequencies were analysed using the tool Kmasker (Schmutzer *et al*., 2014) and exported into BED format. Screening of BAC assemblies against TREP (Version 10) (Wicker *et al*., 2002) was performed with megaBLAST (Zhang *et al*., 2000) and the BLAST output also converted into BED format. The commands ‘merge’ and ‘intersect’ from the BEDtools suite (Quinlan and Hall, 2010) were used to extract and combine features and to compare them to aligned sequences of the barley WGS contigs (International Barley Genome Sequencing *et al*., 2012).

## Supporting information


**Figure S1** Analysis of contig/scaffold L50 as well as gain of longest sequence per BAC and reduction of amount of sequences in relation to contig L50.
**Figure S2** Structure of the sequenced DNA fragments.

**Table S1** Accession numbers of 668 BAC assemblies generated in this study and associated raw read data.

**Table S2** Accession number of reference BACs.

**Table S3** Mapped mate pair reads per BAC.

**Table S4** Assembly statistics for 668 BAC assemblies (paired‐end assemblies and scaffolded assemblies).
**Table S5** Assembly quality statistics for sequenced reference BACs (paired‐end assemblies prior to scaffolding).

**Table S6** Sequencing indices for multiplex BAC sequencing.
**Data S1** Detailed description of web‐lab methods.
