# Supplementary material for: Multiplex sequencing of bacterial artificial chromosomes for assembling complex plant genomes
Source: Plant Biotechnol J. 2016 Jan 23;14(7):1511–22. doi: 10.1111/pbi.12511 (PMC5066668; doi:10.1111/pbi.12511)
Supplement: Supplementary file 1 — Figure S1 Analysis of contig/scaffold L50 as well as gain of longest sequence per BAC and reduction of amount of sequences in relation to contig L50. Figure S2 Structure of the sequenced DNA fragments. [file PBI-14-1511-s005.pptx]

## Slide 1
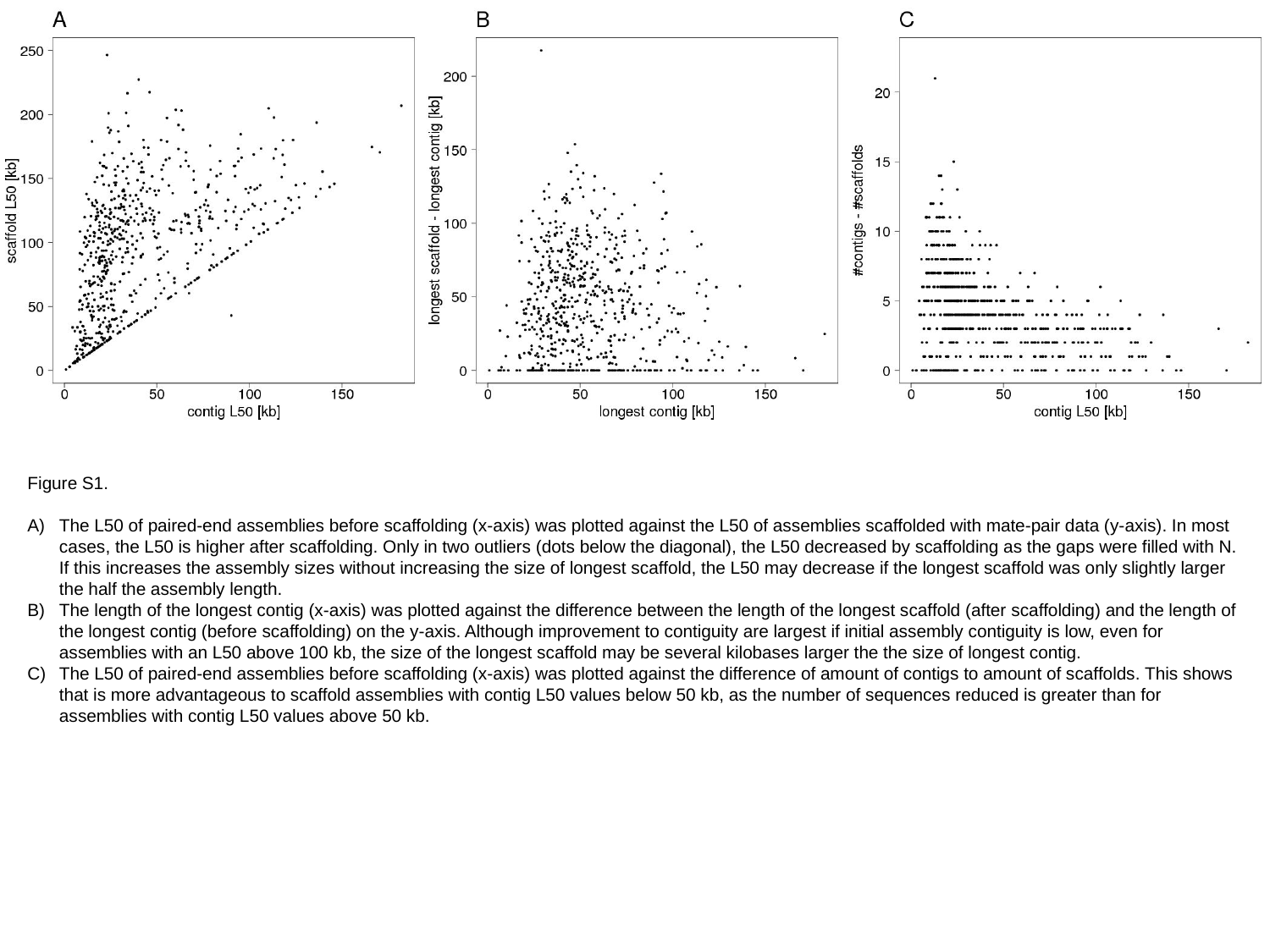

Figure S1.
The L50 of paired-end assemblies before scaffolding (x-axis) was plotted against the L50 of assemblies scaffolded with mate-pair data (y-axis). In most cases, the L50 is higher after scaffolding. Only in two outliers (dots below the diagonal), the L50 decreased by scaffolding as the gaps were filled with N. If this increases the assembly sizes without increasing the size of longest scaffold, the L50 may decrease if the longest scaffold was only slightly larger the half the assembly length.
The length of the longest contig (x-axis) was plotted against the difference between the length of the longest scaffold (after scaffolding) and the length of the longest contig (before scaffolding) on the y-axis. Although improvement to contiguity are largest if initial assembly contiguity is low, even for assemblies with an L50 above 100 kb, the size of the longest scaffold may be several kilobases larger the the size of longest contig.
The L50 of paired-end assemblies before scaffolding (x-axis) was plotted against the difference of amount of contigs to amount of scaffolds. This shows that is more advantageous to scaffold assemblies with contig L50 values below 50 kb, as the number of sequences reduced is greater than for assemblies with contig L50 values above 50 kb.

## Slide 2
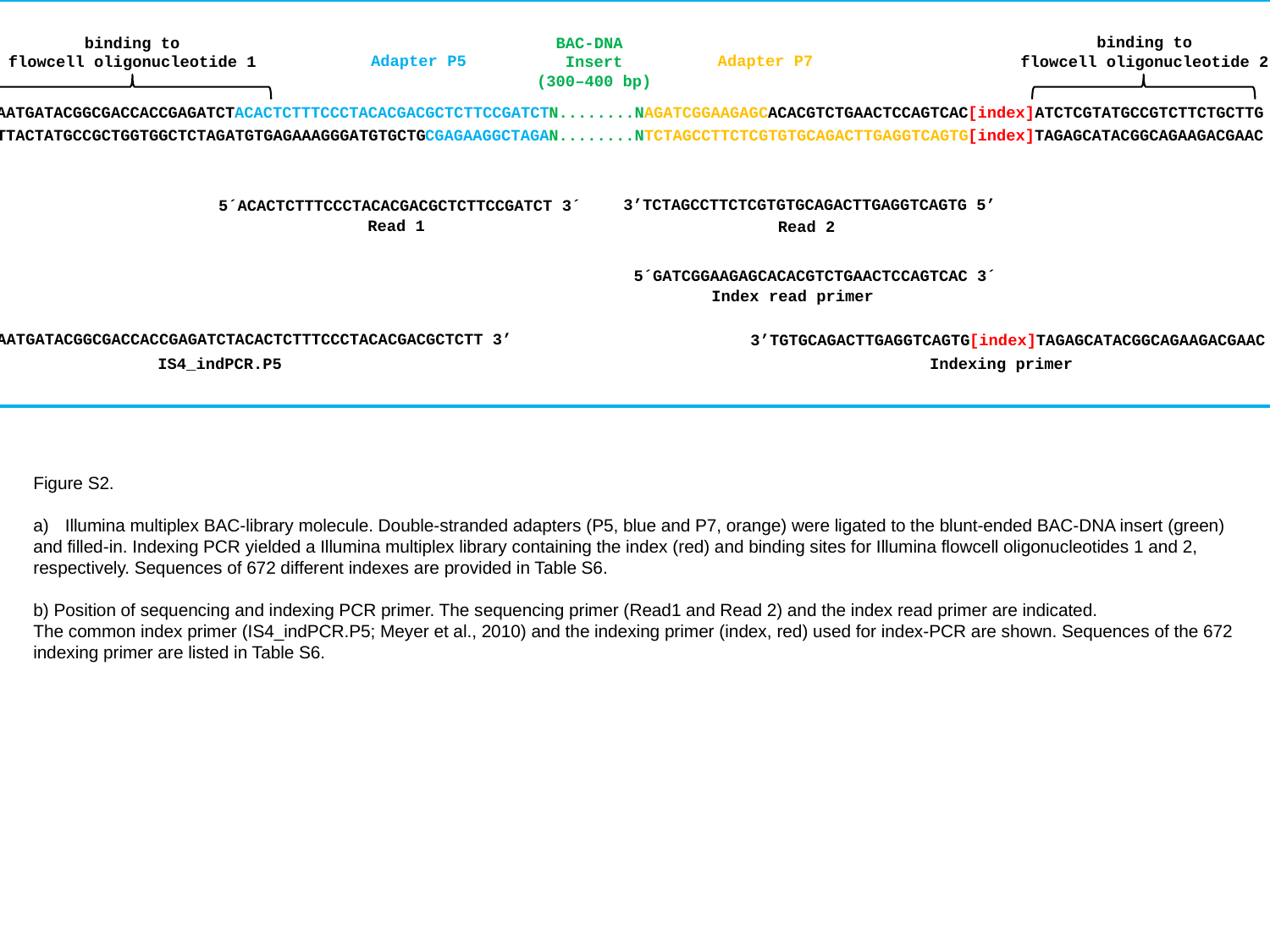

a)
binding to
flowcell oligonucleotide 2
binding to
flowcell oligonucleotide 1
BAC-DNA
Insert
(300–400 bp)
Adapter P5
Adapter P7
5´AATGATACGGCGACCACCGAGATCTACACTCTTTCCCTACACGACGCTCTTCCGATCTN........NAGATCGGAAGAGCACACGTCTGAACTCCAGTCAC[index]ATCTCGTATGCCGTCTTCTGCTTG 3´
3´TTACTATGCCGCTGGTGGCTCTAGATGTGAGAAAGGGATGTGCTGCGAGAAGGCTAGAN........NTCTAGCCTTCTCGTGTGCAGACTTGAGGTCAGTG[index]TAGAGCATACGGCAGAAGACGAAC 5´
b)
 3’TCTAGCCTTCTCGTGTGCAGACTTGAGGTCAGTG 5’
5´ACACTCTTTCCCTACACGACGCTCTTCCGATCT 3´
Read 1
Read 2
5´GATCGGAAGAGCACACGTCTGAACTCCAGTCAC 3´
Index read primer
5´AATGATACGGCGACCACCGAGATCTACACTCTTTCCCTACACGACGCTCTT 3’
 3’TGTGCAGACTTGAGGTCAGTG[index]TAGAGCATACGGCAGAAGACGAAC 5’
Indexing primer
IS4_indPCR.P5
Figure S2.
Illumina multiplex BAC-library molecule. Double-stranded adapters (P5, blue and P7, orange) were ligated to the blunt-ended BAC-DNA insert (green)
and filled-in. Indexing PCR yielded a Illumina multiplex library containing the index (red) and binding sites for Illumina flowcell oligonucleotides 1 and 2,
respectively. Sequences of 672 different indexes are provided in Table S6.
b) Position of sequencing and indexing PCR primer. The sequencing primer (Read1 and Read 2) and the index read primer are indicated.
The common index primer (IS4_indPCR.P5; Meyer et al., 2010) and the indexing primer (index, red) used for index-PCR are shown. Sequences of the 672
indexing primer are listed in Table S6.
