# Supplementary material for: Multiplex sequencing of bacterial artificial chromosomes for assembling complex plant genomes
Source: Plant Biotechnol J. 2016 Jan 23;14(7):1511–22. doi: 10.1111/pbi.12511 (PMC5066668; doi:10.1111/pbi.12511)
Supplement: Supplementary file 3 — Table S2 Accession number of reference BACs. [file PBI-14-1511-s001.docx]

**Supplementary Data**

**Table S2. Accession number of reference BACs.**

| BAC | Accession Number | Organism |
| --- | --- | --- |
| NCBI_AF252830_25 | AF252830 | Homo sapiens |
| NCBI_AF252830_26 | AF252830 | Homo sapiens |
| NCBI_AF252830_27 | AF252830 | Homo sapiens |
| NCBI_AF252830_28 | AF252830 | Homo sapiens |
| NCBI_AF252830_29 | AF252830 | Homo sapiens |
| NCBI_AF252830_30 | AF252830 | Homo sapiens |
| NCBI_AF285443_23 | AF285443 | Homo sapiens |
| NCBI_AF285443_24 | AF285443 | Homo sapiens |
| NCBI_AF285443_25 | AF285443 | Homo sapiens |
| NCBI_AF285443_26 | AF285443 | Homo sapiens |
| NCBI_AF285443_27 | AF285443 | Homo sapiens |
| NCBI_AF285443_28 | AF285443 | Homo sapiens |
| NCBI_AF285443_29 | AF285443 | Homo sapiens |
| NCBI_AF285443_30 | AF285443 | Homo sapiens |
| HVVMRXALLhA0184G09 | AY268139 | Hordeum vulgare |

## Table S5. Assembly quality statistics for sequenced reference BACs (paired-end assemblies prior to scaffolding)

| BAC | L50 | No. of misassemblies | No. of local misassemblies | Indels | Genome fraction^1^ | No. of contigs |
| --- | --- | --- | --- | --- | --- | --- |
| NCBI_AF252830_25 | 28,436 | 1 | 21 | 1 | 98.8 | 10 |
| NCBI_AF252830_26 | 42,065 | 1 | 25 | 0 | 98.3 | 10 |
| NCBI_AF252830_27 | 33,568 | 0 | 21 | 0 | 99.2 | 10 |
| NCBI_AF252830_28 | 22,969 | 1 | 22 | 4 | 99.2 | 10 |
| NCBI_AF252830_29 | 24,767 | 1 | 23 | 0 | 98.5 | 12 |
| NCBI_AF252830_30 | 33,219 | 1 | 16 | 4 | 99.0 | 10 |
| NCBI_AF285443_23 | 22,966 | 1 | 21 | 4 | 99.2 | 9 |
| NCBI_AF285443_24 | 28,212 | 2 | 6 | 5 | 97.8 | 11 |
| NCBI_AF285443_25 | 19,571 | 2 | 16 | 8 | 99.0 | 10 |
| NCBI_AF285443_26 | 22,973 | 3 | 16 | 3 | 98.7 | 11 |
| NCBI_AF285443_27 | 17,305 | 1 | 14 | 21 | 96.4 | 12 |
| NCBI_AF285443_28 | 16,594 | 4 | 15 | 7 | 98.0 | 12 |
| NCBI_AF285443_29 | 33,856 | 0 | 12 | 5 | 99.1 | 6 |
| NCBI_AF285443_30 | 23,003 | 2 | 20 | 10 | 99.3 | 8 |
| HVVMRXALLhA0184G09 | 42,330 | 0 | 8 | 5 | 97.1 | 7 |
| mean | 27,456 | 1 | 17 | 5 | 98.5 | 10 |

## ^1^ Fraction of the genomic insert that is represented in the assembly

## Supplementary Note 1

## BAC DNA isolation

The alkaline lysis method (Birnboim and Doly, 1979) was optimised for the high-throughput isolation of BAC DNA from *E. coli*. Briefly, bacterial cultures were grown (37°C, 20 h) under agitation (220 rpm) in 1.6 ml 2 x YT liquid medium (Sambrook et al., 2001) supplemented with chloramphenicol (12.5 µg/ml) using 2 ml polypropylene 96-deepwell-plates (Ritter riplate, natural; #3845-43001-0020) covered with gas-permeable foil (Macherey-Nagel, #740675). In order to obtain sufficient BAC DNA, duplicates of cultures were inoculated. Cells were harvested by centrifugation (swing-out rotor; 4 °C, 20 min, 3,800 g). The supernatant was removed completely, and pellets were frozen for at least 30 min at -20°C or stored overnight (-20°C). Pellets were resuspended at room temperature (RT) in 200 µl solution I (50 mM Tris-Cl, 10 mM EDTA, 0.5 mg/ml RNase A, pH 8.0) and lysed by adding 200 µl solution II (0.2 M NaOH, 1% (w/v) SDS). The suspension was inverted gently five times and incubated for 5 min on ice. After the addition of 200 µl ice-cold solution III (Sambrook et al., 2001) the sample was inverted gently five times and incubated for 10 min on ice. The cell debris was removed by centrifugation (swing-out rotor; 4 °C, 30 min, 4,800 x g). The clear supernatant (520 µl) was transferred into a new 96-well plate. DNA was precipitated by adding 350 µl Isopropanol. The samples were inverted three times, and the DNA was collected by centrifugation (swing-out rotor; 20 °C, 30 min, 4,000 g). The supernatant was discarded, and the DNA was washed by the addition of 400 µl 70% ethanol. Samples were centrifuged (swing-out rotor; 20 °C, 15 min, 4,000 g), the supernatant was removed and the DNA was air-dried (45 min, 37°C). The DNA was dissolved overnight under agitation (RT) in 100 µl TE (pH 7.5). Duplicates of the BAC DNA were pooled and stored in sealed 0.5 ml polypropylene 96-well plates (Nunc, #267245) at -20°C until used for sequencing library production.

## Preparation of shotgun sequencing libraries

Shotgun sequencing of a pool of 668 individually barcoded BACs (Illumina multiplex library) was performed essentially as described previously (Meyer and Kircher, 2010). For fragmentation, 54 µl BAC-DNA (100 pg to 1 µg) were pipetted into 96 microTUBE plates (Covaris) and subjected to focused acoustic energy (Covaris E220 instrument: 450 W ultrasonic power, 30% duty factor, 200 cycles per burst, 100 sec treatment time). Following fragmentation, BAC DNA fragments (50 µl, size range 300 - 400 bp) were transferred into 96-well plates. For DNA clean-up in the 96-well plate setup, solid-phase reversible immobilisation (SPRI) involving carboxyl-modified magnetic beads was employed. DNA was precipitated by adding 1.8 volumes (90 µl) of the SPRI-bead suspension (MagNa beads) (Himmelbach et al., 2014). The samples were mixed and incubated (5 min, RT). Beads containing the DNA were reclaimed using a DynaMag-96 Side Skirted Magnetic Particle Concentrator (MPC_96_, Invitrogen, #120.27). After 2 min incubation, the clear supernatant was discarded. Beads were washed twice with 150 µl 70% ethanol for 30 sec while placed in the MPC_96_ and dried completely (20 min, RT). For elution the beads were suspended in 52 µl EBT (10 mM Tris-Cl, 0.05% Tween 20, pH 8.5), incubated for 1 min (RT) and reclaimed using a MPC_96_. After 2 min incubation the supernatant (50 µl) was transferred into new 96-well plates. Blunt-end repair of DNA involving T4 DNA polymerase and T4 polynucleotide kinase and SPRI purification was performed as described elsewhere (Himmelbach et al., 2014). In order to monitor the subsequent enzymatic reactions, a positive and a blank control were included (Meyer and Kircher, 2010). At random DNA samples were tested for the size distribution using an Agilent 2100 Electrophoresis Bioanalyzer (Agilent, #G2939AA) and a High Sensitivity DNA Kit (Agilent, #5067-4626). The size peak of the samples was between 300 and 400 bp. Using T4 DNA ligase the blunt-end repaired DNA was provided with adapter P5 and adapter P7 and SPRI purified as described elsewhere (Himmelbach et al., 2014). Adapter ligated DNA was filled-in using *Bst* DNA polymerase (large fragment) and SPRI purified as described (Himmelbach et al., 2014). The performance of the reactions was verified by electrophoresis (2% agarose gel) of the positive and blank controls side-by-side with the untreated positive control (Himmelbach et al., 2014). The success of adapter ligations was monitored by testing in parallel for a band shift (+67 bp) compared to the non-ligated control of the test fragment. Indexing PCR using 20 – 100 ng template DNA and reaction clean-up (SPRI) of the PCR-products was essentially performed as described elsewhere (Himmelbach et al., 2014). Here, a set of 672 different index primers (Table S3) was created according to the previously published guidelines (Meyer and Kircher, 2010) and used for barcoding individual BAC samples. Following an initial denaturation at 98°C (30 s), the DNA amplification was performed for 16 cycles (98°C for 10 s, 60°C for 20 s, and 72°C for 20 s). After a final extension (72°C for 10 min) the products were stored at 8°C. The quality and quantity of randomly selected PCR products was controlled by standard agarose gel electrophoresis (Himmelbach et al., 2014). In order to obtain a comparable number of sequence reads for each individual BAC, differently barcoded BAC samples were pooled in equimolar manner. To achieve this, each purified PCR-product was quantified in quadruplicates using a Synergy HT Multi-Mode Microplate Reader (BioTek), software Gen 5 2.00 (BioTek) and the Quant-iT^TM^ PicoGreen dsDNA Assay Kit (invitrogen, P11496). Briefly, the purified PCR-products were diluted 500-fold in TE, and 50 µl of the dilution were pipetted into a black 384 well polypropylene microplate (Greiner bio-one, #781209). Samples were mixed with 50 µl PicoGreen dye (1000-fold diluted in TE), and the fluorescence was measured (excitation: 485 nm, emission: 528 nm). The sample DNA concentration was determined based on a standard curve using lambda DNA (New England Biolabs). Six nanograms of each purified PCR-product were pooled and mixed thoroughly. The pooled library was size-selected using agarose gel electrophoresis (Himmelbach et al., 2014). DNA was revealed using SYBR-Gold dye (Life Technologies, #S11494) and excitated by visible blue light emitted from a Dark Reader blue light transilluminator (Clare Chemical Research, # DR46B). The region of interest was excised between 420 and 520 bp and purified as described earlier (Himmelbach et al., 2014). The average size of the shotgun BAC library was determined electrophoretically using an Agilent 2100 Bioanalyzer and a High Sensitivity DNA Chip. Precautions to prevent contaminations during library construction were as described (Himmelbach et al., 2014). The structure of the adaptor-ligated DNA fragments is provided as Figure S1. Libraries were quantified using Real-Time PCR essentially as described previously (Mascher et al., 2013). Here, a dilution series of an Illumina multiplex reference library covering the range between 10 pM and 1 fM (standard curve) was employed. The sequencing library was diluted to 10 nM and used in cluster formation on an Illumina (San Diego, CA) cBot. Paired end sequencing (one lane with up to 672 BACs) was performed using Illumina`s HiSeq 2000 instrument. Cluster formation, 2 x 100 cycles paired end sequencing-by-synthesis and index read (8 cycles) were performed according to protocols provided by the manufacturer (Illumina). Sequences were extracted by CASAVA version 1.8.2 (GenomeAnalysis-Pipeline).

## Preparation of Nextera mate-pair sequencing libraries

For the generation of Nextera mate-pair sequencing libraries non-fragmented and non-barcoded DNA of the previously paired-end sequenced BACs were distributed into two equally sized pools and quantified using the Qubit 2.0 fluorometer (Thermo Fisher Scientific, #Q32871). Libraries were prepared following the instructions of the manufacturer (Illumina, FC-132-1001). Briefly, tagmentation was performed using 3 µg pooled BAC-DNA, 40 µl Tagment Buffer Mate Pair and 6 µl Mate Pair Tagment Enzyme (200 µl total volume). Following incubation (55°C, 30 min) DNA was purified as described (Illumina, FC-132-1001). For strand displacement 30 µl tagmented DNA, 10 µl 10x strand displacement buffer, 4 µl dNTPs and 5 µl Strand Displacement Polymerase (100 µl total volume) were mixed and incubated (20°C, 30 min). The products were precipitated by the addition of 50 µl AMPure XP beads (Beckman Coulter, #A63882) and purified as described (Illumina, FC-132-1001). Size selection using agarose gels, purification of large DNA fragments (7 kbp to 10 kbp range), circularization and digestion of linear DNA were according to the manual (Illumina, FC-132-1001). The circularized DNA was sheared using adaptive focused acoustic energy (Covaris S220 AFA Ultrasonicator: 240 W ultrasonic power, 20% duty factor, 200 cycles per burst, 40 sec treatment time) and Covaris T6 glass tubes. Affinity purification of mate-pairs using streptavidin magnetic beads, end-repair, A-tailing, adapter ligation, PCR-amplification and purification of the PCR-products were performed as described elsewhere (Illumina, FC-132-1001). The average size of the mate-pair BAC library was determined electrophoretically using an Agilent 2100 Bioanalyzer and a High Sensitivity DNA Kit (Agilent, #5067-4626). Libraries were quantified using Real-Time PCR essentially as described previously (Mascher et al., 2013). Here, a dilution series of a Nextera mate-pair reference library covering the range between 10 pM and 1 fM (standard curve) was used. The sequencing library was diluted to 10 nM and used for cluster formation on an Illumina (San Diego, CA) cBot for paired-end sequencing-by-synthesis with Illumina’s HiSeq 2000 (2 x 100 cycles). In routine experiments up to 12 libraries provided with different indices (each constructed from 384 BACs) were sequenced in one HiSeq 2000 lane. In addition, libraries were sequenced using the Illumina MiSeq (2 x 250 cycles) according to protocols provided by the manufacturer (Illumina Inc.). In one MiSeq run, up to six barcoded libraries (384 BACs each) were sequenced in parallel. FASTQ sequence files were extracted with CASAVA version 1.8.2 (HiSeq2000) or with the MiSeqReporter Software (MiSeq).

**Supplementary References**

**Birnboim H, Doly J** (1979) A rapid alkaline extraction procedure for screening recombinant plasmid DNA. Nucleic Acids Res **7:** 1513-1523

**Himmelbach A, Knauft M, Stein N** (2014) Plant Sequence Capture Optimised for Illumina Sequencing. Bio-protocols **4:** e1166

**Mascher M, Richmond TA, Gerhardt DJ, Himmelbach A, Clissold L, Sampath D, Ayling S, Steuernagel B, Pfeifer M, D'Ascenzo M** (2013) Barley whole exome capture: a tool for genomic research in the genus Hordeum and beyond. The Plant Journal **76:** 494-505

**Meyer M, Kircher M** (2010) Illumina sequencing library preparation for highly multiplexed target capture and sequencing. Cold Spring Harb Protoc **2010:** pdb prot5448

**Sambrook J, Russell DW, Russell DW** (2001) Molecular cloning: a laboratory manual (3-volume set). Cold Spring Harbor Laboratory Press, Cold Spring Harbor, New York
